# Somatic *RUNX1* exonic deletions in myelodysplastic syndrome: A rare yet high‐risk recurrent genetic aberration

**DOI:** 10.1002/hem3.70452

**Published:** 2026-08-21

**Authors:** Ahmed Al‐Djaber, Albin Österroos, Alessandro Baldi, Arielle R. Munters, Nikolaj Juul Nitschke, Klas Raaschou‐Jensen, Claudia Schöllkopf, Marianne Tang Severinsen, Tatjana Pandzic, Manja Meggendorfer, Joachim Weischenfeldt, Anne Stidsholt Roug, Kirsten Grønbæk, Claes Ladenvall, Torsten Haferlach, Claudia Haferlach, Thale Kristin Olsen, Panagiotis Baliakas

**Affiliations:** ^1^ Department of Immunology, Genetics and Pathology Uppsala University Uppsala Sweden; ^2^ Department of Molecular Diagnostics and Clinical Genetics Uppsala University Hospital Uppsala Sweden; ^3^ MLL Munich Leukemia Laboratory Munich Germany; ^4^ Department of Hematology Rigshospitalet, Copenhagen University Hospital Copenhagen Denmark; ^5^ Biotech Research and Innovation Centre (BRIC), Faculty of Health and Medical Sciences University of Copenhagen Copenhagen Denmark; ^6^ Department of Haematology Odense University Hospital Odense Denmark; ^7^ Department of Hematology Clinical Cancer Research Center, Aalborg University Hospital Aalborg Denmark; ^8^ Department of Clinical Medicine Aalborg University Aalborg Denmark; ^9^ Department of Hematology Aarhus University Hospital Aarhus Denmark; ^10^ Department of Clinical Medicine, Faculty of Health and Medical Sciences University of Copenhagen Copenhagen Denmark

Myelodysplastic syndromes (MDS) are a group of clonal hematopoietic disorders characterized by significant genetic and clinical heterogeneity.^1^, ^2^, ^3^ Risk stratification and therapeutic decision‐making are increasingly guided by the underlying genomic landscape.^4^, ^5^ Mutations in the runt‐related transcription factor 1 (*RUNX1*) gene are observed in 10%–15% of all de novo MDS cases and are among the most well‐established prognostic molecular markers in MDS and myeloid neoplasms (MNs).^6^, ^7^, ^8^, ^9^, ^10^, ^11^ RUNX1 is a transcription factor essential for normal hematopoiesis, acting as a master regulator of hematopoietic stem cell maintenance, lineage commitment, and megakaryopoiesis.^12^, ^13^ Most *RUNX1* mutations cause loss‐of‐function and are associated with specific clinico‐biological features.^14^

We recently reported that somatic *RUNX1* exonic deletions (RUNX1^del^) are recurrent and associated with a poor prognosis in acute myeloid leukemia (AML).^15^ In addition, the spectrum of reported germline *RUNX1* mutations includes a substantial proportion of gross and intragenic exonic deletions, likely underdiagnosed in the past due to technical limitations.^16^, ^17^, ^18^ Prompted by these facts, we hypothesized that somatic RUNX1^del^ may be present in MDS.

We analyzed a cohort of 1299 MDS patients using a comprehensive diagnostic approach that included whole‐genome sequencing (WGS, *n* = 263), array comparative genomic hybridization (CGH, *n* = 584), and an in‐house next‐generation sequencing (NGS) panel (*n* = 85) or a combination of the three methods (*n* = 367), allowing reliable detection of RUNX1^del^. Methodological details are described in Supporting Information S1: Supplementary Methods.

Patient characteristics are described in Supporting Information S2: Table 1. Twenty‐five (1.9%) patients had *RUNX1* deletions (RUNX1^del^), 121 (9.3%) had *RUNX1* mutations (RUNX1^mut^), and 1153 (88.8%) had no *RUNX1* aberration (RUNX1^wt^). Three of the RUNX1^mut^ patients also had a concurrent *RUNX1* deletion. The final cohort had a median age of 73 years (range 23–94 years) with 756 (58.2%) men.

Diagnostic categorization by WHO 2017^19^ was available for 1242 (95.6%) patients (Supporting Information S2: Table 1). RUNX1^del^ and RUNX1^mut^ patients (40.0% and 44.6%, respectively) had a significantly higher prevalence of MDS with excess blasts 2 (MDS‐EB‐2) compared to RUNX1^wt^ patients (17.6%, P = 0.01 for RUNX1^del^ vs. RUNX1^wt^, P < 0.001 for RUNX1^mut^ vs. RUNX1^wt^). MDS with loss of 5q (MDS 5q‐) and MDS with ring sideroblasts (MDS‐RS) were underrepresented within *RUNX1* aberrant patients when compared to RUNX1^wt^ patients, with the difference being more profound between RUNX1^mut^ versus RUNX1^wt^ (5.4% vs. 12.1%, P = 0.03 and 9% vs. 22.4%, P < 0.001, respectively).

RUNX1^del^ patients had significantly lower diagnostic hemoglobin (median 85 vs. 97 vs. 100 g/L, P < 0.001 for RUNX1^del^ vs. RUNX1^wt^, P = 0.002 for RUNX1^del^ vs. RUNX1^mut^) and platelets (median 39 vs. 91 vs. 148 ×10^9^/L, P < 0.001 for RUNX1^del^ vs. RUNX1^wt^, P = 0.03 for RUNX1^del^ vs. RUNX1^mut^) compared to RUNX1^mut^ and RUNX1^wt^ patients. Bone marrow blast percentages were higher in RUNX1^del^ and RUNX1^mut^ patients compared to RUNX1^wt^ patients (median 8.5% and 8.0% vs. 4.0%, P = 0.001 for RUNX1^del^ vs. RUNX1^wt^, P < 0.001 for RUNX1^mut^ vs. RUNX1^wt^) (Supporting Information S2: Table 1).

Cytogenetic data (≥20 analyzed metaphases) were available for 1223 (94.1%) patients. Normal cytogenetics was significantly less frequent in RUNX1^del^ than in RUNX1^mut^ patients (12.0% vs. 52.9%, P < 0.001) and in RUNX1^wt^ patients (12.0% vs. 45.2%, P = 0.002), mainly driven by a significantly higher proportion of patients with a complex karyotype (>3 abnormalities) in RUNX1^del^ patients compared to RUNX1^mut^ and RUNX1^wt^ patients (54.2% vs. 1.9% vs. 13.9%, all P < 0.001).

This translated into differences in risk stratification per the Comprehensive Cytogenetic Scoring System (CCSS)^20^ (Figure 1A). Only 8.3% of RUNX1^del^ patients were classified at the “good” risk tier compared to 58.5% and 58.4% for RUNX1^mut^ and RUNX1^wt^ patients, respectively (P < 0.001 for RUNX1^del^ vs. RUNX1^mut^ or RUNX1^wt^). Conversely, patients at the “very poor” risk tier were more common among RUNX1^del^ patients compared to RUNX1^mut^ and RUNX1^wt^ patients (54.2% vs. 1.9% vs. 11.1%, all P < 0.001).

**Figure 1:**
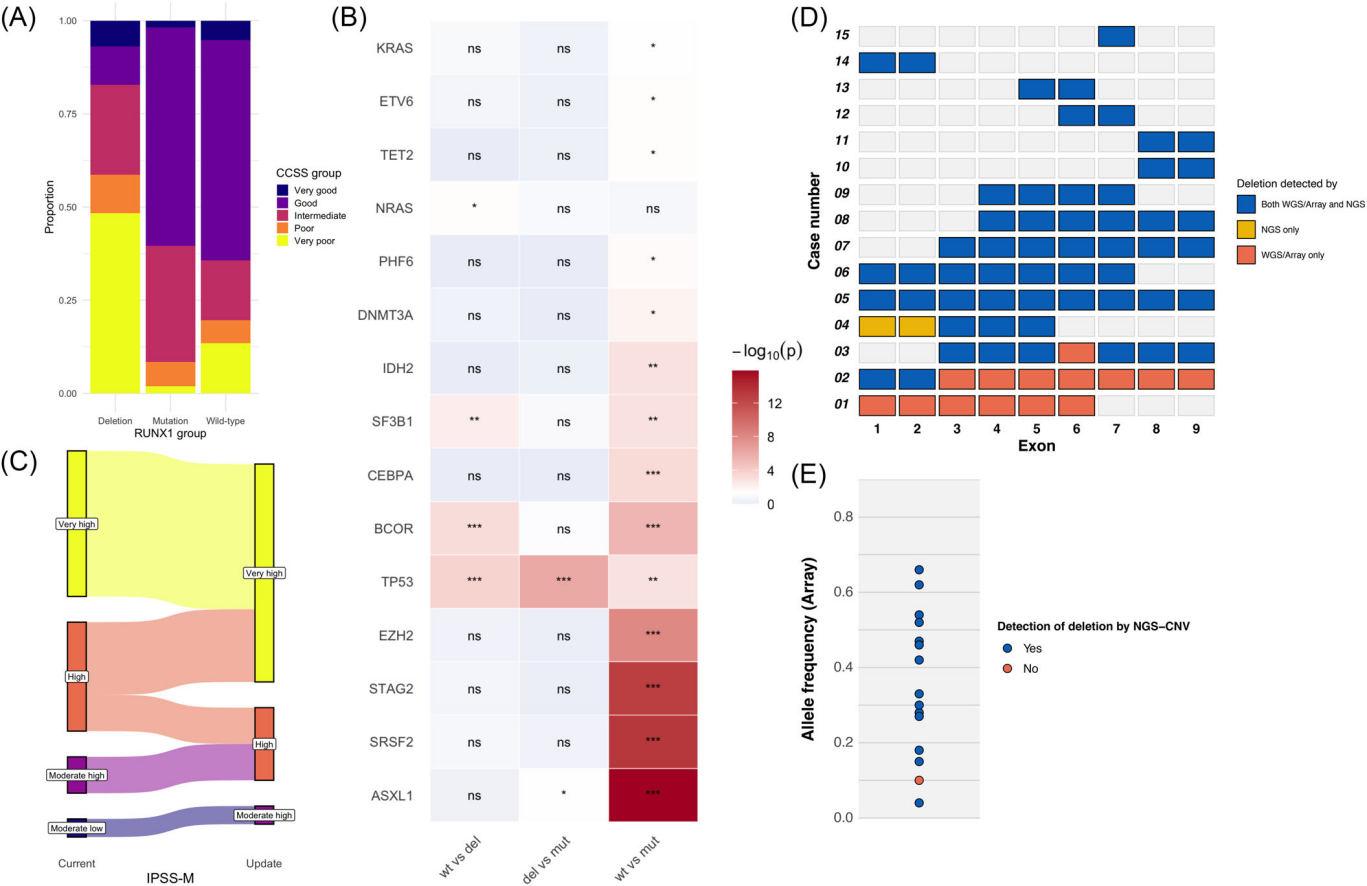
**Molecular characteristics per *RUNX1* status and assessment of *RUNX1* status with a targeted next‐generation sequencing (NGS) panel. (A)** Stacked bar chart illustrating the relative proportions per *RUNX1* status and stratified by the Comprehensive Cytogenetic Scoring System (CCSS). Data are presented as percentages of the total within each group. **(B)** Heatmap illustrating associations between *RUNX1* status and the presence of co‐occurring mutations where the top 15 genes are ranked by statistical significance. Colors represent the −log _10_ transformed P‐values. Raw P‐values from Fisher's exact tests are denoted by ***P < 0.001, **P < 0.01, and *P < 0.05, and are available in Supporting Information S1: Table 2. **(C)** Sankey diagram illustrating the migration of risk classifications for a subset of patients (*n* = 17) with available data for restratification by the Molecular International Prognostic Scoring System (IPSS‐M) model. The visualization shows the upward migration of several patients into higher clinical risk categories when *RUNX1* genomic deletions are reclassified as functionally equivalent to *RUNX1* mutations. **(D)** Brick plot showing exonic deletions across the *RUNX1* gene (Exons 1–9) in 15 confirmed cases with *RUNX1* deletion detected by array or whole‐genome sequencing (WGS). Deletions detected by both WGS/array and NGS are shown in blue, deletions detected by NGS only in yellow, and deletions undetected by NGS in orange. **(E)** Scatter plot of allele frequencies from array‐based data for the detected deletions in the 15 cases. Blue dots represent cases where deletions were detected by our in‐house NGS‐based copy number variation (CNV)‐approach. The deletions not detected by NGS are represented in orange. del, deletion; mut, mutation; ns, not significant; wt, wild‐type.

Data on co‐mutations were available for 1151 (88.6%) patients (Figure 1B, Supporting Information S1: Table 2). The average number of somatic mutations per patient was 1.1 (range 0–7) with no inter‐group differences per *RUNX1* status concerning mutational burden.

*TP53* mutations were more common in RUNX1^del^ than RUNX1^mut^ patients (55.6% vs. 5.4%, P < 0.001). Comparative analyses of co‐mutations between RUNX1^del^ and RUNX1^wt^ revealed higher mutation frequencies in RUNX1^del^ patients for *BCOR* (42.9% vs. 2.5%, P < 0.001), *NRAS* (20.0% vs. 2.9%, P = 0.04), and *TP53* (55.5% vs. 16.5%, P < 0.001). Mutations in *SF3B1* were less commonly seen in RUNX1^del^ compared to RUNX1^wt^ (0% vs. 29.8%, P = 0.005) (Figure 1B, Supporting Information S1: Table 2). After correction for multiple testing, differences remained statistically significant for *BCOR* (adj.P = 0.01) and *TP53* (adj.P = 0.007).

In the RUNX1^del^ group, IPSS‐M data were available for 17 patients. We compared IPSS‐M scores with and without the *RUNX1* deletion counting as a *RUNX1* aberration (Figure 1C). Based on baseline IPSS‐M scores (excluding RUNX1^del^), patient risk categories “very high” (*n* = 8), “high” (*n* = 6), “moderate high” (*n* = 2), and “moderate low” (*n* = 1). Accounting for RUNX1^del^ upstaged 6 patients (35%) to a higher risk tier: 3 from “high” to “very high,” 2 from “moderate high” to “high” and 1 from “moderate low” to “moderate high.”

For validation, our in‐house NGS‐based CNV approach was evaluated on 116 samples (including an independent cohort of 26 AML patients), of which 15 were orthogonally confirmed positive for RUNX1^del^ via array and/or WGS. The NGS panel detected 14 of 15 cases (93%), fully resolving 11 deletions and partially identifying 3 (Figure 1D). Deletion sizes ranged from single‐exon (*n* = 1) to whole‐gene (*n* = 2). For whole‐gene deletions, karyotype and array data ruled out monosomy 21 or larger macro‐deletions. A representative exonic deletion (Exons 4–7) is illustrated in Supporting Information S1: Figure S1.

We observed no false‐positive deletions (specificity 100% [95% confidence interval (CI) 63%–100%], sensitivity 93% [95% CI 68%–100%]). Allele frequencies in array data for the 15 RUNX1^del^ ranged from 0.04 to 0.66 across the samples, with the single undetected deletion occurring in a sample with an allele frequency of 0.1 (Figure 1E).

We assessed the clinical impact regarding *RUNX1* status. There were 200 patients (18.1%) in the whole cohort that transformed into AML (median follow‐up time 6.2 years, range 0–16.9). The risk of AML transformation was similar between patients with RUNX1^del^ or RUNX1^mut^ and significantly higher than in RUNX1^wt^ patients (41.2% vs. 42.3% vs. 14.9%) (Figure 2A). In a multivariable logistic regression analysis assessing the risk of transformation to AML, there was no independent significance of RUNX1^del^ (odds ratio [OR] 1.46, 95% CI 0.38–4.63) (Figure 2E). RUNX1^mut^ and higher diagnostic bone marrow blast percentage were associated with a higher transformation risk.

**Figure 2:**
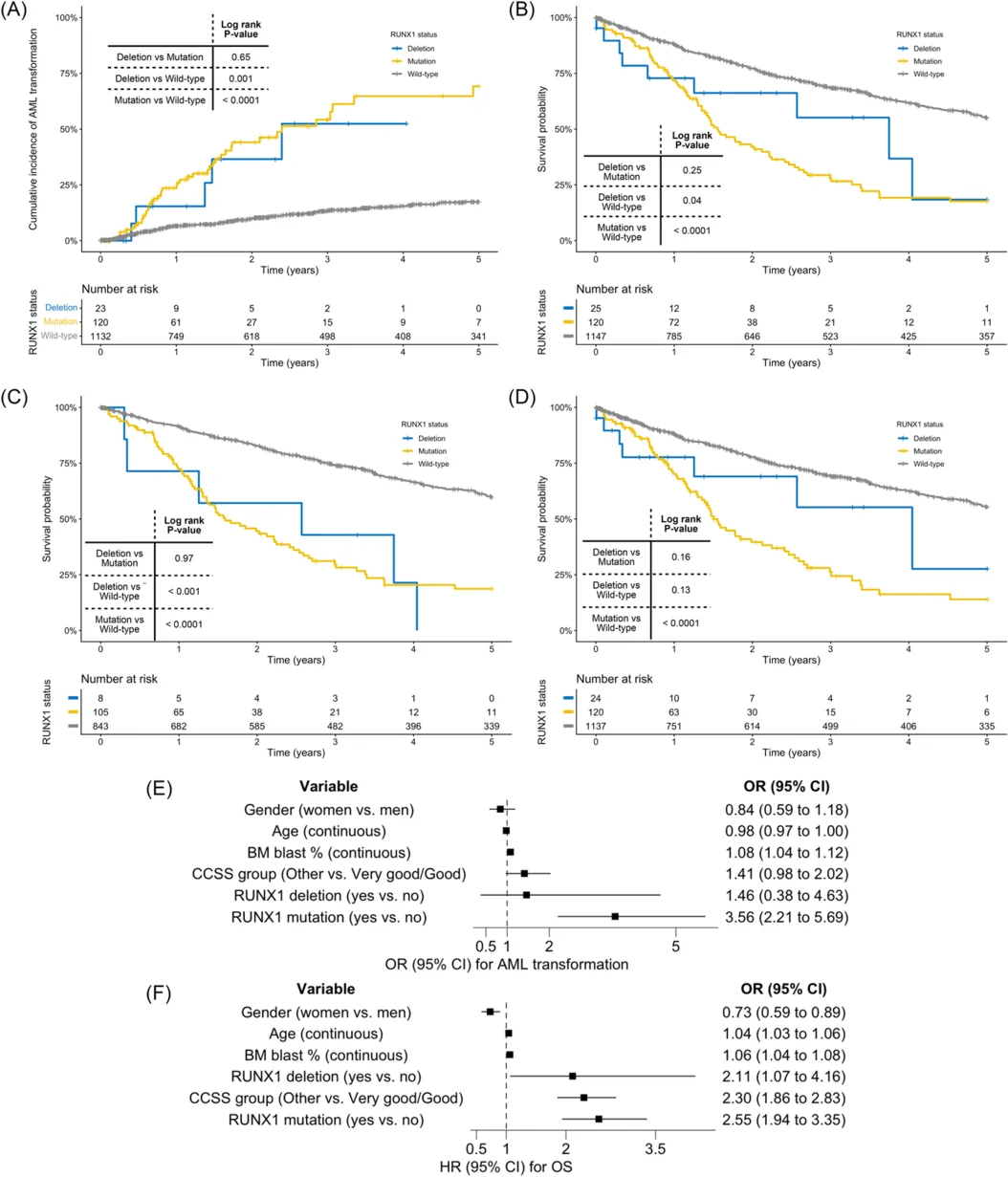
**Prognostic impact of** ***RUNX1*** **deletions. (A)** Kaplan–Meier plot illustrating the 5‐year cumulative incidence of leukemic transformation by *RUNX1* status. **(B–C)** Kaplan–Meier plot for 5‐year overall survival (OS) per *RUNX1* status in (B) with exclusion of *TP53*‐positive cases in (C). **(D)** Kaplan–Meier plot for OS applying censoring at the time of allogeneic stem cell transplantation. Statistical differences between the curves were assessed using the log‐rank test with Benjamini–Hochberg adjustment for pairwise comparisons. **(E)** Forest plot displaying multivariable‐adjusted odds ratio (OR) for leukemic transformation. Predictors were assessed using logistic regression. **(F)** Forest plot displaying multivariable‐adjusted hazard ratios (HRs) for 5‐year OS. Estimates were derived from a Cox proportional hazards model. All error bars represent 95% confidence intervals (CIs), and the dashed vertical line indicates the null effect (OR/HR = 1.0). AML, acute myeloid leukemia; BM, bone marrow; CCSS, Comprehensive Cytogenetic Scoring System.

RUNX1^del^ patients had a median overall survival (OS) of 3.7 years (95% CI 1.3–NA) compared to 1.5 years (95% CI 1.4–2.2) for RUNX1^mut^ patients (P = 0.25). The median OS for RUNX1^wt^ patients extended beyond the maximum follow‐up time of 5 years with significantly worse OS in RUNX1^del^ and RUNX1^mut^ patients (P = 0.04 for RUNX1^del^ vs. RUNX1^wt^; P < 0.0001 for RUNX1^del^ vs. RUNX1^wt^) (Figure 2B). The differences in OS remained after excluding *TP53* mutations (P < 0.001 RUNX1^del^ vs. RUNX1^wt^; P = 0.97 RUNX1^del^ vs. RUNX1^mut^) (Figure 2C).

Eighty patients (12.3%) in the whole cohort underwent allo‐SCT. When we applied censoring at allo‐SCT, the median OS in RUNX1^del^ patients was 4.0 years (95% CI 1.3–NA) while the median OS for RUNX1^mut^ and RUNX1^wt^ patients remained unchanged (log‐rank P = 0.16 and P = 0.13, respectively) (Figure 2D).

In a multivariable Cox proportional hazards model evaluating OS, the presence of RUNX1^del^ was independently associated with poorer OS (hazard ratio [HR] 2.11, 95% CI 1.07–4.16). RUNX1^mut^ was also associated with poorer OS (HR 2.55, 95% CI 1.94–3.35) (Figure 2F). Male gender, higher age, higher bone marrow blast percentage, and “intermediate,” “poor,” and “very poor” CCSS risk groups were all predictors of poorer OS in our cohort.

In summary, we report a ~2% incidence of RUNX1^del^ in MDS, notably lower than in AML (~25%). RUNX1^del^ was associated with an unfavorable clinical outcome, which remained significant even after excluding *TP53* aberrations. Given that RUNX1^del^ is likely underdiagnosed and associated with shorter OS, re‐evaluating these aberrations across MNs is warranted to clarify their disease specificity. Reliable detection is increasingly critical as *RUNX1*‐targeted therapies enter clinical evaluation. We successfully integrated a CNV backbone into our routine diagnostic NGS panel, validating it as a reliable, cost‐effective solution that fits into standard clinical workflows.

We cannot exclude a potential germline origin in identified RUNX1^del^ patients as we only analyzed blood or bone marrow, though patients lacked a history of pre‐diagnostic thrombocytopenia or inherited blood disorders. While the study's retrospective design is a limitation, it is uniquely powered by the largest unselected MDS cohort to date mapping the prevalence and clinical impact of RUNX1^del^.

As somatic RUNX1^del^ represents a rare but recurrent, high‐risk entity in MDS, we advocate for updating current diagnostic pipelines to capture these aberrations using targeted NGS solutions or comprehensive sequencing methodologies.

## AUTHOR CONTRIBUTIONS

**Ahmed Al‐Djaber**: Methodology; data curation; investigation; formal analysis; visualization; writing—original draft; writing—review and editing; validation; project administration. **Albin Österroos**: Methodology; data curation; investigation; validation; formal analysis; visualization; writing—original draft; writing—review and editing; project administration. **Alessandro Baldi**: Data curation; investigation; formal analysis; writing—review and editing; methodology. **Arielle R. Munters**: Data curation; investigation; writing—review and editing; formal analysis; validation. **Nikolaj Juul Nitschke**: Data curation; investigation; writing—review and editing. **Klas Raaschou‐Jensen**: Data curation; investigation; writing—review and editing. **Claudia Schöllkopf**: Data curation; investigation; writing—review and editing. **Marianne Tang Severinsen**: Data curation; investigation; writing—review and editing. **Tatjana Pandzic**: Methodology; data curation; investigation; validation; formal analysis; writing—review and editing. **Manja Meggendorfer**: Methodology; data curation; investigation; formal analysis; writing—review and editing. **Joachim Weischenfeldt**: Data curation; investigation; writing—review and editing. **Anne Stidsholt Roug**: Data curation; investigation; writing—review and editing. **Kirsten Grønbæk**: Data curation; investigation; writing—review and editing. **Claes Ladenvall**: Methodology; software; data curation; investigation; validation; formal analysis; writing—review and editing. **Torsten Haferlach**: Methodology; data curation; investigation; formal analysis; writing—review and editing. **Claudia Haferlach**: Data curation; investigation; methodology; formal analysis; writing—review and editing. **Thale Kristin Olsen**: Conceptualization; methodology; data curation; investigation; validation; formal analysis; visualization; project administration; writing—original draft; writing—review and editing. **Panagiotis Baliakas**: Conceptualization; methodology; data curation; investigation; validation; formal analysis; supervision; funding acquisition; project administration; resources; writing—original draft; writing—review and editing.

## CONFLICT OF INTEREST STATEMENT

T.H. and C.H. declare part ownership of MLL Munich Leukemia Laboratory. A.B. and M.M. are employed by the MLL Munich Leukemia Laboratory. The remaining authors declare no relevant conflicts of interest.

## ETHICS STATEMENT

This study was approved by the local Regional Ethical Committee (Dnr 2023‐00987‐01) in accordance with the Declaration of Helsinki, including written consent.

## FUNDING

A.A.‐D. received support from ALF Kansliet Uppsala (LUL‐990593). P.B. received support from ALF Kansliet Uppsala (ALF‐990591, ALF‐1002575, and ALF‐991381, Swedish Society of Medicine (SLS‐961271), Lions CancerFonden (2022‐1050146 and 2023‐1050180), Vleugels stiftelse (2020‐01), Jeansson Stiftelser (2023), and Swedish Cancer Society (23 2745 Pj, Projekt). T.K.O. received funding from the Swedish Childhood Cancer Foundation (Barncancerfonden, TJ2021‐0010).

## Supporting information

Supporting Information.

Supporting Information.

## Data Availability

The data that support the findings of this study are available from the corresponding author upon reasonable request.
