# Supplementary material for: Somatic RUNX1 exonic deletions in myelodysplastic syndrome: A rare yet high‐risk recurrent genetic aberration
Source: Hemasphere. 2026 Aug 21;10(8):e70452. doi: 10.1002/hem3.70452 (PMC13495559; doi:10.1002/hem3.70452)
Supplement: Supplementary file 1 [file HEM3-10-e70452-s001.docx]

**Supplementary Material**

**Somatic *RUNX1* exonic deletions in myelodysplastic syndrome: a rare yet high-risk recurrent genetic aberration**

**Supplementary Methods**

*Patient cohort*

The present cohort included 1299 well-characterized unselected patients with MDS, diagnosed and treated between 2005 and 2022. Exclusion criteria were lack of diagnostic sample (n = 195), lack of clinical data (n = 2) and other suspected/confirmed diagnoses than MDS (n = 31) (**Supplementary Figure 1**). DNA was extracted from diagnostic bone marrow or peripheral blood samples. *RUNX1* aberrations were detected with array CGH (n = 584), whole-genome sequencing (n = 263), our NGS-based in-house panel (n = 85) or a combination of the three methods (n = 367). Cytogenetic analysis was performed in 1245 samples, of which 1223 could be classified according to the Comprehensive Cytogenetic Scoring System (CCSS), included in IPSS-M. Sufficient information for IPSS-M stratification was available for 17 RUNX^del^ patients. This study was approved by the local Regional Ethical Committee (Dnr 2023-00987-01) in accordance with the Declaration of Helsinki, including written consent.

*Genetic analysis*

Chromosome preparations and banding analysis of bone marrow and/or peripheral blood samples were performed as previously described according to standard methods ^1^. We used the guidelines from the International System for Human Cytogenomic Nomenclature for classification of abnormalities and karyotypes ^2^. Array CGH was performed as previously described ^3^. Whole Genome Sequencing (WGS) (median coverage: 100x) was performed as described below. At Uppsala University Hospital, diagnostic assessment of MN was performed using a customized, clinically validated NGS panel ^4^. In brief, the design includes 199 target genes and includes a backbone of structural normal variants (SVs) across the genome for copy number variation (CNV) detection. CNVs were detected using two callers; CNVkit (v0.9.9) ^5^ and GATK (v4.1.7.0) ^6^. In this study, we re-designed the panel with enrichment of the backbone in the *RUNX1* region to optimize the detection of exonic and larger *RUNX1* deletions, after first validating a *RUNX1* targeted design ^4^. As part of the validation, a total of 116 samples were analysed with the panel, of which 26 originated from a separate cohort of AML patients. A subset of the validation cohort was sequenced using a localized panel restricted to chromosome 21. While the capture design for the targeted regions was identical to the full NGS panel, the limited genomic backbone necessitated a small modification of the interpretation of the CNV calls. Interpretation of NGS-derived copy-number profiles was performed blinded to the corresponding array/WGS findings to avoid observer bias.

*Statistical analysis*

Fisher’s exact test (for categorical variables) and the Mann–Whitney U test (for continuous variables) were used for group comparisons regarding clinical and molecular parameters. For co-mutations, comparisons were restricted to patients with available sequencing data for each gene. Patients without sequencing data were excluded from that specific analysis and were not assumed to be wild-type.

Time to AML transformation was measured from the date of MDS diagnosis until final follow-up or time to AML transformation. Overall survival (OS) was measured from the date of MDS diagnosis until final follow-up or death from any cause. The predetermined maximal follow-up time was 5 years and patients alive at the final follow-up were censored. Median follow-up was estimated using the reverse Kaplan–Meier method. Curves for OS and time to AML transformation were constructed using the Kaplan–Meier method and the log-rank test was used for group comparisons. Log rank P-values adjusted for multiple comparisons applying the Benjamini-Hochberg (BH) procedure. OS analyses were performed both with and without censoring at the time of allogeneic stem cell transplantation (allo-SCT). Multivariable logistic and Cox regression models were utilized to estimate the impact of *RUNX1* status on time to AML transformation and OS while adjusting for gender, age at diagnosis, bone marrow blasts at diagnosis and cytogenetic risk group per CCSS.

All statistical analyses were two sided with P < 0.05 considered as statistically significant. All statistical analyses were performed using the computing environment R version 4.0.2 ^7^ with the following packages: Baseline characteristic tables and comparative descriptive statistics were generated using gtsummary (version 2.5.0) ^8^ and compareGroups (version 4.10.2) ^9^. For time-to-event analyses, we utilized the packages survival (version 3.8-6) ^10^ and survminer (version 0.5.1). Data visualization relied on ggplot2 (version 4.0.2) ^11^, ComplexHeatmap (version 2.22.0) ^12^, ggsankey (version 0.0.99999) ^13^ and forestploter (version 1.1.3).

*Whole Genome Sequencing (WGS)*

Whole Genome Sequencing (WGS) libraries were prepared from 1 µg of DNA with the TruSeq PCR free library prep kit following the manufacturer’s recommendations (Illumina, San Diego, CA, USA) and 2x150bp paired-end sequences were generated on a NovaSeq 6000 or HiSeqX instrument with 100x coverage (Illumina, San Diego, CA, USA). Reads were aligned to the human reference genome (GRCh37, Ensembl annotation) using the Isaac aligner (v3.16.02.19) ^14^ through BaseSpace’s WGS app (v5, Illumina, San Diego, CA, USA) with default parameters. Resulting BAM files were used in BaseSpace’s Tumor/Normal app (v3) to call single nucleotide variants (SNV) and small indels (<50bp) with Strelka (v2.4.7) ^15^ and large-scale structural variants (SV) with Manta (v0.28.0) ^16^. As no sample specific normal tissue was available, a so-called unmatched normal was used in its place to reduce technical artefacts and germline calls. For this reason, WGS was performed on gender-matched genomic DNA from a mixture of multiple anonymous donors (Promega, Fitchburg, WI, USA). To further remove potential germline variants, each SNV/small indel was queried against the gnomAD database (v2.1.1) ^17^ and variants with global population frequencies >0.05% where excluded. Further analysis was performed on protein-altering and splice-site variants only. CNVs were called with GATK (v4.0.8.1) ^6^ using the Broad Institute’s recommended best practices pipeline. Here, two panels of normals (PON) were used for denoising consisting of 124 female and 191 male samples which presented a normal karyotype during routine diagnostics.

**Supplementary Methods: References**

1.	Schoch C, Schnittger S, Bursch S, et al. Comparison of chromosome banding analysis, interphase- and hypermetaphase-FISH, qualitative and quantitative PCR for diagnosis and for follow-up in chronic myeloid leukemia: a study on 350 cases. *Leukemia*. 2002;16(1):53-59. doi:10.1038/sj.leu.2402329

2.	*ISCN 2016: An International System for Human Cytogenomic Nomenclature*. 2016.

3.	Stengel A, Kern W, Meggendorfer M, et al. Number of RUNX1 mutations, wild-type allele loss and additional mutations impact on prognosis in adult RUNX1-mutated AML. *Leukemia*. 2018;32(2):295-302. doi:10.1038/leu.2017.239

4.	Al-Djaber A, Munters A, Cammenga J, et al. Low Rate of Germline Investigation for Variants of Suspected Germline Origin Detected During the Diagnostic Work-Up of Myeloid Neoplasms. *EJHaem*. 2025;6(6):e70206. doi:10.1002/jha2.70206

5.	Talevich E, Shain AH, Botton T, Bastian BC. CNVkit: Genome-Wide Copy Number Detection and Visualization from Targeted DNA Sequencing. *PLoS Comput Biol*. 2016;12(4):e1004873. doi:10.1371/journal.pcbi.1004873

6.	McKenna A, Hanna M, Banks E, et al. The Genome Analysis Toolkit: a MapReduce framework for analyzing next-generation DNA sequencing data. *Genome Res*. 2010;20(9):1297-1303. doi:10.1101/gr.107524.110

7.	R: A language and environment for statistical computing. 2024. Accessed May 12, 2026. https://www.r-project.org/

8.	Sjoberg D, Whiting K, Curry M, Lavery J, Larmarange J. Reproducible summary tables with the gtsummary package. *The R Journal*. 2021;13:570-580.

9.	Subirana I, Sanz H, Vila J. Building bivariate tables: The compareGroups package for R. *Journal of Statistical Software*. 2014;57(12):1-16.

10.	Therneau TM, Grambsch PM. *Modeling Survival Data: Extending the Cox Model*. Springer; 2000.

11.	Wickham H. *Elegant Graphics for Data Analysis*. Springer; 2016. https://ggplot2.tidyverse.org

12.	Gu Z, Eils R, Schlesner M. Complex heatmaps reveal patterns and correlations in multidimensional genomic data. *Bioinformatics*. 2016;32(18):2847-2849. doi:10.1093/bioinformatics/btw313

13.	Sjoberg D. ggsankey: Sankey, Alluvial and Sankey Bump Plots. https://github.com/davidsjoberg/ggsankey

14.	Raczy C, Petrovski R, Saunders CT, et al. Isaac: ultra-fast whole-genome secondary analysis on Illumina sequencing platforms. *Bioinformatics*. 2013;29(16):2041-2043. doi:10.1093/bioinformatics/btt314

15.	Kim S, Scheffler K, Halpern AL, et al. Strelka2: fast and accurate calling of germline and somatic variants. *Nat Methods*. 2018;15(8):591-594. doi:10.1038/s41592-018-0051-x

16.	Chen X, Schulz-Trieglaff O, Shaw R, et al. Manta: rapid detection of structural variants and indels for germline and cancer sequencing applications. *Bioinformatics*. 2016;32(8):1220-1222. doi:10.1093/bioinformatics/btv710

17.	Karczewski KJ, Francioli LC, Tiao G, et al. The Mutational Constraint Spectrum Quantified from Variation in 141,456 Humans. Genomics; 2019. doi:10.1101/531210

**Supplementary Figure 1**

**Flowchart of included patients.**

Abbreviations: MLL: Munich Leukemia Laboratory, MDS: myelodysplastic syndrome.

**Supplementary Figure 2**

***RUNX1* copy number variation (CNV) analysis.** The top panel shows the CNV profile across the *RUNX1*-gene in a normal sample and a *RUNX1*-deleted sample. Deletions are indicated by a reduced log2 ratio across the affected region, with loss of exon 4-7 in the *RUNX1*-deleted sample. The bottom panel shows a schematic representation of exon structure of the *RUNX1*-normal and the *RUNX1*-deleted samples.

**Supplementary Table 2.** Co-mutation patterns per RUNX1 status.

| **Supplementary Table 1: Co-mutation patterns per RUNX1 status.** | | | | | | | |
| --- | --- | --- | --- | --- | --- | --- | --- |
|  | **Total** | ***RUNX1* wild-type (wt)** | ***RUNX1* deletion (del)** | ***RUNX1* mutation (mut)** | **P** | **P** | **P** |
|  | **N=1 151** | **N=1017** | **N=18** | **N=120** | **(wt vs del)** | **(wt vs mut)** | **(del vs mut)** |
| *ASXL1* | 222 (19.8%) | 160 (16.1%) | 3 (21.4%) | 59 (52.2%) | 0.484 | <0.001 | 0.088 |
| *BCOR* | 39 (3.88%) | 22 (2.46%) | 3 (42.9%) | 14 (13.3%) | 0.001 | <0.001 | 0.070 |
| *CBL* | 43 (4.03%) | 35 (3.70%) | 2 (20.0%) | 6 (5.45%) | 0.162 | 0.428 | 0.201 |
| *CEBPA* | 16 (2.11%) | 9 (1.34%) | 0 (0.00%) | 7 (8.97%) | 1.000 | 0.002 | 1.000 |
| *DNMT3A* | 146 (13.8%) | 139 (14.8%) | 0 (0.00%) | 7 (6.36%) | 0.560 | 0.068 | 1.000 |
| *ETNK1* | 15 (1.80%) | 14 (1.89%) | 0 (0.00%) | 1 (1.18%) | 1.000 | 1.000 | 1.000 |
| *ETV6* | 27 (2.47%) | 20 (2.06%) | 1 (8.33%) | 6 (5.50%) | 0.344 | 0.117 | 0.528 |
| *EZH2* | 56 (5.08%) | 34 (3.47%) | 1 (8.33%) | 21 (18.8%) | 0.528 | <0.001 | 0.691 |
| *FLT3* | 10 (1.00%) | 7 (0.78%) | 1 (11.1%) | 2 (1.94%) | 0.232 | 0.237 | 0.237 |
| *GATA2* | 16 (1.87%) | 14 (1.85%) | 0 (0.00%) | 2 (2.27%) | 1.000 | 1.000 | 1.000 |
| *GNB1* | 2 (0.27%) | 2 (0.30%) | 0 (0.00%) | 0 (0.00%) | 1.000 | 1.000 | . |
| *IDH1* | 27 (2.52%) | 21 (2.21%) | 0 (0.00%) | 6 (5.45%) | 1.000 | 0.157 | 1.000 |
| *IDH2* | 39 (3.64%) | 28 (2.95%) | 0 (0.00%) | 11 (10.0%) | 1.000 | 0.004 | 0.894 |
| *JAK2* | 47 (4.36%) | 43 (4.50%) | 0 (0.00%) | 4 (3.64%) | 1.000 | 1.000 | 1.000 |
| *KIT* | 9 (1.03%) | 6 (0.77%) | 0 (0.00%) | 3 (3.37%) | 1.000 | 0.167 | 1.000 |
| *KRAS* | 20 (1.88%) | 14 (1.48%) | 1 (10.0%) | 5 (4.50%) | 0.221 | 0.123 | 0.411 |
| *NF1* | 28 (3.03%) | 22 (2.68%) | 1 (14.3%) | 5 (5.15%) | 0.291 | 0.291 | 0.349 |
| *NPM1* | 10 (1.04%) | 10 (1.18%) | 0 (0.00%) | 0 (0.00%) | 1.000 | 1.000 | . |
| *NRAS* | 36 (3.37%) | 28 (2.95%) | 2 (20.0%) | 6 (5.41%) | 0.110 | 0.158 | 0.158 |
| *PHF6* | 23 (2.28%) | 17 (1.90%) | 0 (0.00%) | 6 (5.66%) | 1.000 | 0.083 | 1.000 |
| *PPM1D* | 17 (2.29%) | 16 (2.42%) | 0 (0.00%) | 1 (1.32%) | 1.000 | 1.000 | 1.000 |
| *PRPF8* | 13 (1.42%) | 11 (1.36%) | 0 (0.00%) | 2 (2.08%) | 1.000 | 1.000 | 1.000 |
| *PTPN11* | 13 (1.34%) | 10 (1.17%) | 0 (0.00%) | 3 (2.97%) | 1.000 | 0.447 | 1.000 |
| *RUNX1* | 120 (10.4%) | 0 (0.00%) | 0 (0.00%) | 120 (100%) | . | <0.001 | <0.001 |
| *SETBP1* | 27 (3.18%) | 22 (2.93%) | 1 (10.0%) | 4 (4.55%) | 0.423 | 0.423 | 0.423 |
| *SF3B1* | 316 (28.0%) | 299 (29.8%) | 0 (0.00%) | 17 (15.2%) | 0.007 | 0.005 | 0.127 |
| *SRSF2* | 173 (16.0%) | 121 (12.6%) | 3 (27.3%) | 49 (43.8%) | 0.234 | <0.001 | 0.353 |
| *STAG2* | 58 (6.34%) | 30 (3.69%) | 1 (14.3%) | 27 (28.1%) | 0.356 | <0.001 | 0.671 |
| *TET2* | 330 (30.1%) | 284 (29.1%) | 3 (25.0%) | 43 (39.1%) | 1.000 | 0.119 | 0.798 |
| *TP53* | 184 (16.0%) | 168 (16.5%) | 10 (55.6%) | 6 (5.36%) | <0.001 | 0.003 | <0.001 |
| *U2AF1* | 81 (7.63%) | 68 (7.22%) | 2 (20.0%) | 11 (10.1%) | 0.376 | 0.376 | 0.376 |
| *WT1* | 5 (0.60%) | 5 (0.68%) | 0 (0.00%) | 0 (0.00%) | 1.000 | 1.000 | . |
| *ZRSR2* | 62 (6.29%) | 54 (6.16%) | 0 (0.00%) | 8 (7.92%) | 1.000 | 1.000 | 1.000 |
